# Arabic Translation and Psychometric Validation of PROMIS General Life Satisfaction Short Form in the General Population

**DOI:** 10.3390/healthcare11233034

**Published:** 2023-11-24

**Authors:** Hadeel R. Bakhsh, Nouf S. Aldajani, Bodor Bin Sheeha, Monira I. Aldhahi, Atheer A. Alsomali, Ghada K. Alhamrani, Rahaf Z. Alamri, Rehab Alhasani

**Affiliations:** Department of Rehabilitation Sciences, College of Health and Rehabilitation Sciences, Princess Nourah Bint Abdulrahman University (PNU), Riyadh 11671, Saudi Arabia; hrbakhsh@pnu.edu.sa (H.R.B.); bhbinsheeha@pnu.edu.sa (B.B.S.); mialdhahi@pnu.edu.sa (M.I.A.); 438001636@pnu.edu.sa (R.Z.A.)

**Keywords:** PROMIS, Arabic, Rasch analysis, outcome measure, psychometrics, quality of life

## Abstract

This study aimed to translate the Patient-Reported Outcomes Measurement Information System (PROMIS) General Life Satisfaction Short Form (GLS SF5a) into the Arabic language and psychometrically validate the scale in the general population of Saudi Arabia. The translation processes followed the international recommendations of the FACIT Measurement System. The study was a multicentre cross-sectional study conducted in Riyadh, Saudi Arabia. A total of 657 individuals who were above 18 years of age and able to write and comprehend Arabic completed the GLS SF5a. Rasch analysis was used to evaluate item fit, reliability indices, item difficulty, principal component analysis and local item dependency. WINSTEPS (v. 5.6.0) was used for the analysis. The translation process and cognitive defibring were completed with no issues. The rating scale categories had a disordered threshold. All items, except one, demonstrated a satisfactory fit to the Rasch model. The reliability of the person separation was 0.86. The scale was unidimensional, and no items showed local dependency. Overall, this study confirms the psychometric properties of the Arabic version of the PROMIS GLS SF5a, which can be used as an instrument for measuring general life satisfaction in the general population. Further research is required to explore responsiveness, interpretability and feasibility in the clinical setting.

## 1. Introduction

Life satisfaction is an important element of health that affects different domains of an individual’s life [1]. The American Psychological Association defines life satisfaction as the extent to which a person finds life rich, meaningful, full or of high quality [2]. It is a critical component for measuring quality of life (QOL) and an indicator of subjective well-being, which is a key aspect of mental health [3]. A substantial body of evidence has consistently underscored the strong correlation between the evaluation of life satisfaction and a multitude of pivotal factors. These include health status, manifestation of symptoms associated with depression and anxiety, perception of pain, quality of sleep, economic prosperity, and levels of physical activity [1,4,5,6]. Therefore, assessing and screening life satisfaction aligns with national health policy recommendations, underlining their essential role in comprehensive healthcare evaluations [7].

Patient-reported outcome measures (PROMs) are self-administered questionnaires that assess outcomes and screen for health conditions. The integration of PROMS within the medical sphere yields substantial enhancements in clinician–patient communication and fosters a collaborative environment conducive to shared decision making for treatment processes [8,9]. Consequently, the Patient-Reported Outcome Measurement Information System (PROMIS) was developed to provide valid, reliable and precise PROMs that would outperform traditional instruments and enable improved outcome measures in clinical practice [8,10]. The Patient-Reported Outcome Measurement Information System (PROMIS) has the potential to assess a range of functional abilities in a wide range of patient ages and numerous languages and can be administered in a variety of ways [10]. Furthermore, it facilitates cross-study data comparisons by adapting the item bank for use in multiple countries.

Within the mental health domain, the PROMIS framework offers a set of adult measures designed to evaluate life satisfaction. Specifically, the General Life Satisfaction (GLS) Short Form 5a (SF5a) was used as an assessment tool. Few studies have focused on assessing the construct validity of PROMIS GLS in different populations. Vaughan et al.’s [11] study was the only one that evaluated the psychometric properties of the PROMIS GLS, specifically assessing structural and construct validity among individuals with musculoskeletal pain in Australia. Their study concluded that the GLS SF5 was unidimensional, with acceptable internal consistency and construct validity in this population [11].

Over the last two decades, the use of PROMs in Arab clinical society has progressed, either by developing new PROMs or by translating and adapting existing measures to specific cultural contexts [12,13,14,15]. Consequently, numerous studies conducted among different healthcare practitioners in Saudi Arabia indicate that the implementation of PROMs helps with directing the plan of care, improving patient–clinician communication and providing a patient-centred approach [12,13,14]. All of these facilitate the process of shared decision making, which is considered a key element of patient-centred care [15,16]. In fact, multiple studies have reported improved QOL, better patient and clinician satisfaction and better treatment outcomes through shared decision making [15,17,18].

To the best of our knowledge, no prior studies have translated or assessed the validity and psychometric properties of the PROMIS GLS SF5a in the Arabic language using Rasch analysis. The advantages of the PROMIS tools, and specifically the PROMIS GLS SF5, lie in their efficiency, precision, standardisation and adaptability [19]. Moreover, the adaptive nature of PROMIS assessments helps reduce respondent burden by tailoring the questions to the individual’s level of the measured construct, thereby reducing the number of questions required for assessment [19].

Therefore, this study aimed to provide an Arabic translation and cultural adaptation of the PROMIS GLS SF5a and evaluate its psychometric properties in general populations in Saudi Arabia.

## 2. Materials and Methods

### 2.1. Translation and Cultural-Adaptation Process

#### 2.1.1. Study Team

The Arabic translation team consisted of linguists, translators, proofreaders and cognitive interviewers contracted with FACITtrans (Table 1). The primary focus was to translate items from the FACIT measurement system. All team members were native Arabic speakers, except for the back translator, who was a native English speaker fluent in Arabic. All members of the FACITtrans Arabic translation team met ISO 17100 standards [20] for professional competencies and translation qualifications.

#### 2.1.2. Translation Process

The FACIT translation process included the following stages.

First stage: Forward translations were carried out by two separate professional translators, who were native Arabic speakers and independently translated the source items in English into Arabic. To ensure accuracy and clarity, a third independent translator, who was also a native speaker in Arabic, reviewed and reconciled the two forward translations.

Second stage: Following the reconciliation process, a native English-speaking translator back-translated the provided content. The translator did not have access to the original English sources or item definitions. The purpose of back-translation was to accurately reflect the content of the target language translation without adding or deleting elements. Next, the translation project manager (TPM) performed a thorough comparison between the original and back-translated English versions to identify any discrepancies. 

Third Stage: Three bilingual translation experts conducted a thorough review of the translation history, carefully selected the most suitable translation for each item and, where needed, offered alternative translations. Prior to finalisation, the translation team at FACITtrans diligently reviewed the reviewers’ comments and meticulously analysed their suggested translations for any potential concerns. They subsequently formulated precise questions and comments to aid the Arabic language coordinator in addressing these issues. In addition to providing the final translation, the language coordinator also included a literal back-translation and an improved back-translation.

Fourth and final stage: The comparability of the translated version was assessed by the FACITtrans team in collaboration with the PROMIS Statistical Centre. The quality assurance process included consistency checks with previous translations and between items. If any additional input was needed, the Arabic coordinator was contacted for consultation, and two independent proofreaders meticulously examined all items to identify any spelling and grammatical issues. Subsequently, a reconciliation process was conducted to address and resolve any discrepancies found in the proofreading comments.

#### 2.1.3. Cognitive Debriefing and Pre-Testing

A pilot test was conducted using a convenience sample of 30 native Arabic speakers from the general population to gain an understanding of how individuals comprehend and explain the items. Participants were eligible to participate in the study if they were 18 years or older, native Arabic speakers and were able to provide verbal consent. The questionnaire items were reviewed and tested on at least six individuals from Saudi Arabia, Morocco, Kuwait, Jordan and Egypt. The authors of this study from Princess Nourah University (PNU) in Saudi Arabia conducted the interviews. The authors have extensive knowledge and experience in conducting cognitive interviews and psychometric evaluations of outcome measures. Furthermore, they received training on the study-specific interviewing protocol through teleconferences facilitated by FACITtrans.

Before each administration, the interviewers provided a comprehensive explanation of the study’s purpose and details to the participants. The respondents completed the questionnaire independently. Subsequently, a cognitive debriefing interview was conducted following a specific script. Emphasis was placed on scrutinising the wording of each translated item. Participants were asked to restate the items in their own words, define specific terms and phrases and describe their decision making process when selecting their responses. This process allowed for FACITtrans to assess the linguistic validity and acceptability of the Arabic items.

After the interviews were completed, the interviewers took on the task of transcribing them. They carefully compiled all the opinions and suggestions expressed by the interviewees into a comprehensive, cognitive debriefing report. To assess the interviewees’ comprehension of each item, the TPM meticulously examined their feedback and identified key issues. In instances where revision suggestions were provided, the TPM, in collaboration with the language coordinator, offered final recommendations for modifications or proposed suitable translation solutions. Finally, the TPM submitted the cognitive debriefing report to the PROMIS Statistical Centre for a quality review.

### 2.2. Study Design and Participants

This was a multicentre cross-sectional study. A convenience sample of participants was recruited between March and May 2023 at the Princess Nourah Bint Abdulrahman University, King Fahad Medical City and King Abdullah bin Abdulaziz University Hospital in Riyadh, Saudi Arabia. The targeted sample size was 729 participants, based on the calculation using Cα with a power calculation of 80% and accounting for a 10% drop-out rate [21]. 

The inclusion criteria were as follows: ability to read and comprehend the Arabic language, healthy 18 years or older, a history of a chronic condition. In this study, the presence of a chronic condition was self-identified by respondents based on the options provided in the first section of the questionnaire. Chronic condition options included metabolic diseases, cardiac diseases, pulmonary diseases, psychological diseases, musculoskeletal diseases, neurological diseases and cancer. The exclusion criteria comprised the presence of cognitive impairments that would interfere with completing the questionnaire and the inability to comprehend the Arabic language.

**Figure 1:**
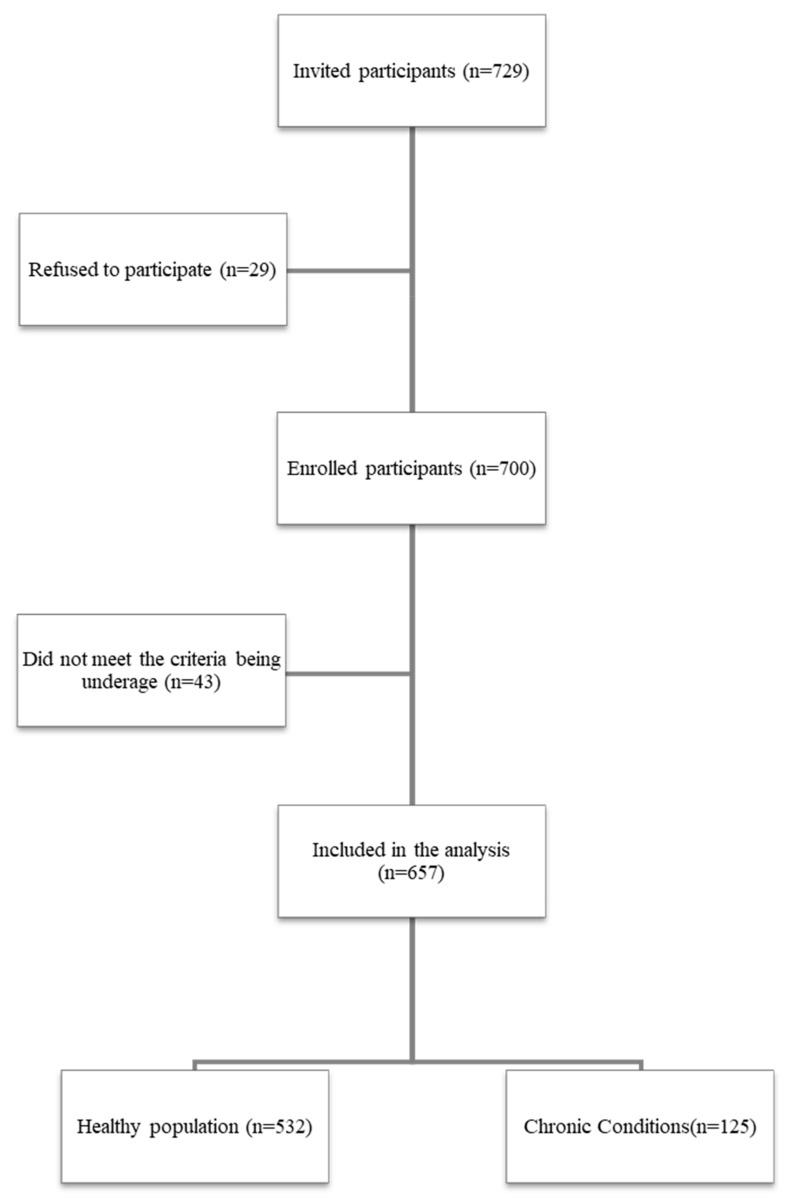
Participants recruitment Process.

A total of 729 participants were invited to participate in this study, of whom 29 (4%) declined to participate and 43 (6%) did not meet the inclusion criteria. For the final analysis, 657 participants (90%) were enrolled. A study recruitment flowchart is shown in Figure 1.

### 2.3. Procedure

Participants were approached by the study team, either in the community at college lobbies and libraries (PNU) or patients waiting in the rehabilitation department at the hospitals mentioned above. Once the participants gave consent for participation, a computerised questionnaire was administered using Microsoft Forms during the data collection process in the hospitals, while a paper-and-pencil format was administered across the hospitals. 

The authors obtained the required licences and authorisation from the PROMIS Health Organization (PHO) in April 2022 to translate the PROMIS GLS item bank into Arabic. The study was approved by the Institutional Review Board of PNU (KACST, KSA: HAP-01-R-059), KAAUH (RO-2023-P-019) and KFMC (H-01-R-012). This study was conducted in accordance with the Declaration of Helsinki.

### 2.4. Measures

Participants completed a survey that consisted of two parts. The first part asked participants to provide sociodemographic information including age, sex, height, weight, educational level, employment status, marital status and place of residence. The second part consisted of the PROMIS GLS SF5a [21]. The estimated completion time ranged from 5 to 7 min.

The PROMIS GLS SF5 was used to assess life satisfaction, with five items rated on a 7-point Likert scale (1 = strongly disagree, 7 = strongly agree). The stem of the question was ‘Indicate how much you agree or disagree’. The lowest raw score was 5, and the highest was 35. The PROMIS uses a *t*-score metric to calculate and interpret responses, which is a standardised measure of the total score for a particular measure with a mean of 50 and a standard deviation (SD) of 10 for a reference population, in which case the US general population is our reference population [22]. 

### 2.5. Data Analysis

Descriptive statistics, including frequency, percentage, mean and standard deviation (SD), were used to identify demographics. Rasch analysis (RA) is a statistical modelling technique that is used primarily to evaluate the relationships between individuals’ abilities and the difficulty of items on a scale [23]. It assesses the probability of a person endorsing or succeeding at an item based on their underlying trait or ability and the item’s inherent difficulty. This method transforms categorical data into interval-level measurements, facilitating more precise comparisons between individuals and items while ensuring measurement reliability and validity. Its application aids in the development of reliable and valid assessment tools and enhances the understanding of latent traits or abilities within a population [23,24]. The following multistage approach for Rasch analysis was applied to assess the PROMIS GLS SF5a metrics using Winsteps^®^ software (v. 5.6.1) [25] using a rating scale model.

Rating scale category functioning was evaluated using the criteria proposed by Linacre (1999) and Wolfe and Smith (2007) to verify the ordered response thresholds for each question (the threshold being the transition point between adjacent categories) [25,26,27]. 

Principal component analysis of the standardised residuals (PCAr) was used to evaluate the following: if other variables are likely to be included in the residuals, the Rasch factor should explain ≥ 50% of the variance. If the eigenvalue of the first residual component (first contrast) is greater than 2, additional factors are likely to be present. The presence of local dependency ‘correlation’ of two items with a correlation greater than 0.30 is regarded as a possible indicator of local dependency.

The internal construct validity of the PROMIS GLS Arabic version was evaluated by assessing how closely the data matched the Rasch model in terms of suitability. For each item on each scale, chi-square fit statistics (expressed as infit and outfit mean-square statistics, MnSq; expectation 1, range 0 to infinity) were calculated. Acceptable fit was defined based on our sample size as mean square values ranging from 0.7 to 1.3, characterised by a standardised *z*-value (ZStd) of less than ±2.0 [26]. To consider an item a misfit, both the MnSq and ZStd values must be outside their specified ranges.

The estimates of item difficulty and subject ability were calculated in logit units, where logit represents the natural logarithm of the odds ratio between mutually exclusive options, such as pass versus fail or higher versus lower responses. Item difficulty refers to the level of difficulty associated with each item, whereas subject ability indicates the location of each individual subject on a common interval scale.

Reliability was evaluated based on both the item and person separation indices, which ranged from 0 to ∞. The item separation index (G) provides an estimate of the standard error units of the spread or separation of items. The reliability of the separation indices represents the degree of confidence in the consistency of the estimates and falls within the range of 0 to 1. Coefficients exceeding 0.80 are considered good, while those surpassing 0.90 are regarded as excellent [23]. 

## 3. Results

### 3.1. Translation and Cultural Adaptation

Thirty adults participated in the cognitive interviews. The participants had an average age of 35.2 years (SD ± 10.5 years). Among the total participants, 50% (*n* = 15) were male. Regarding their educational background, 37% (*n* = 11) had completed high school, 23% (*n* = 7) possessed a diploma certificate and 40% (*n* = 12) had an undergraduate degree. The participants hailed from five different Arab countries, with an equal distribution of 20% (*n* = 5) from Saudi Arabia, Jordan, Egypt, Morocco and Kuwait. All were native Arabic speakers. 

The cognitive debriefing report revealed highly positive feedback concerning the clarity and comprehensibility of the item instructions and response options of the PROMIS GLS. All items were culturally suitable, and the same underlying concept as in the original English was measured. All PROMIS GLS item banks were deemed unequivocally clear, with participants accurately restating the items with precise wording when prompted to do so during the interviews.

### 3.2. Descriptive Statistics

The demographic data of the participants are shown in Table 2. A total of 657 participants had a mean age of 28.44 ± 11.36 years, predominantly female (81.28%). More than half were students (*n* = 287; 43.68%), and the majority were single (70.32%). A total of 125 participants reported having at least one chronic medical condition, with the majority reporting metabolic syndromes (53.60%). The PROMIS GLS SF5a Arabic version had a mean *t*-score of 48.52 ± 10.53 in the general population. 

### 3.3. Rasch Analysis

**Figure 2:**
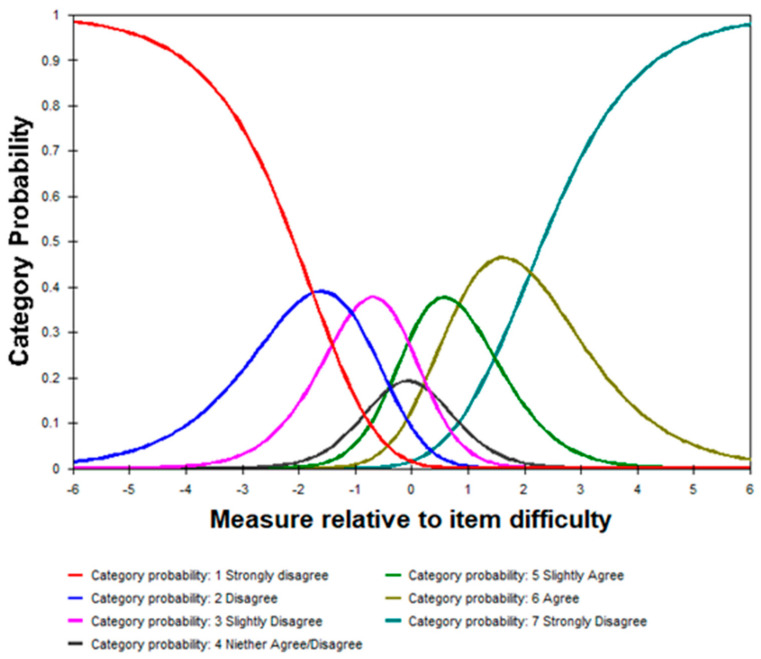
ROMIS General Life Satisfaction SF5a Rating Scale Categories functioning.

*Rating scale diagnostics:* The rating scale of the PROMIS GLS SF5a Arabic version did not meet the criteria for category functioning because of disordered thresholds (Table 3 and Figure 2). 

*Principal component analysis (PCA):* Scale unidimensionality was confirmed by the PCA of standardised residuals, and the variance explained by the Rasch measures was acceptable (72%), whereas that explained by the first contrast in the residuals was low or moderate, with an eigenvalue of 1.4 (8.2%). There was no local dependency on these items (*r* > 0.30).

*Item fit statistics*: Four of the five items fit the Rasch model (see Table 4). Item PA046 (‘If I could live my life over, I would change almost nothing’) somewhat underfitted the module (Outfit MnSq = 1.33), since it had a few unpredictable answers. 

**Figure 3:**
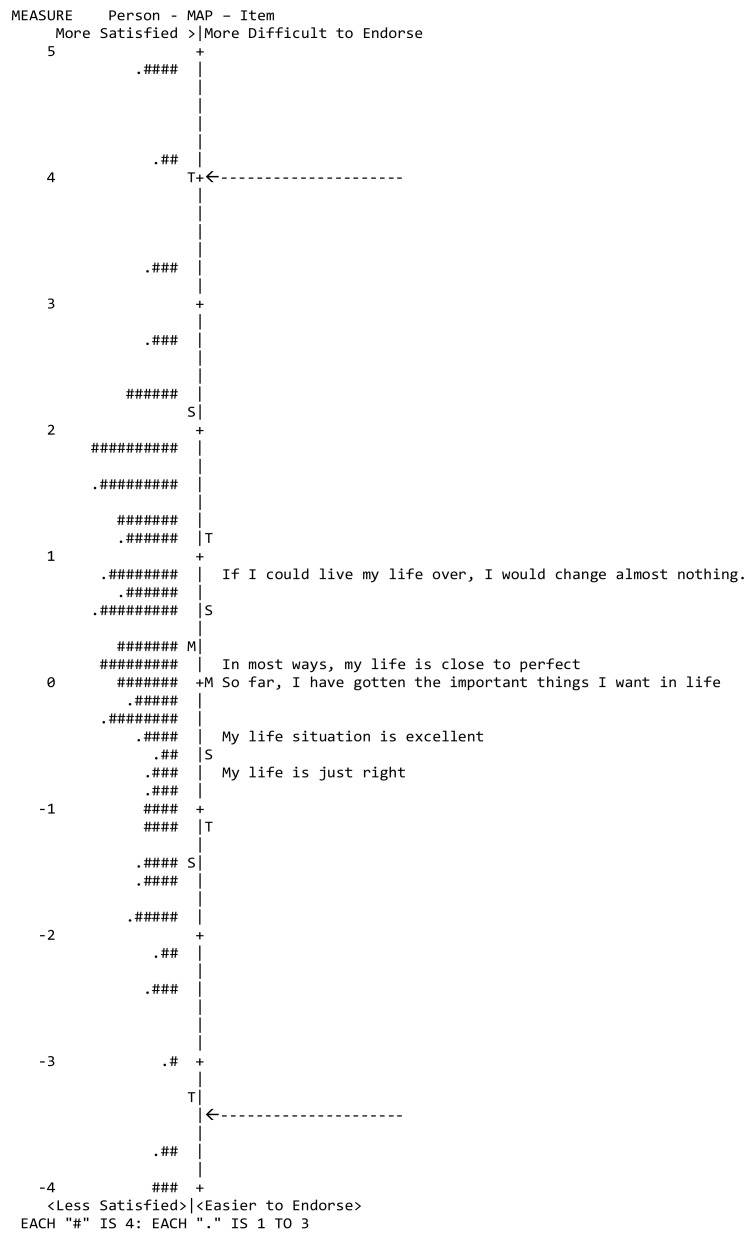
Wright’s Map of participant ability (Satisfaction) and item difficulty Arabic PROMIS General Life Satisfaction SF5a.

*Item difficulty and subject ability*: The range of item difficulty estimates, as demonstrated in Wright’s map (Figure 3), was 0.89 logits. Apart from the five maximum scores, the person’s ability ranged very evenly from 4.08 to −3.70 logits, with an average of 0.29 logits. 

*Reliability*: The person separation index and reliability were 2.45 and 0.86, respectively, whereas the item separation index and reliability were 12.97 and 0.99, respectively. The internal consistency of the PROMIS GLS SF5a Arabic version was excellent (Cronbach’s α = 0.90). 

The vertical line signifies the measure of the variable in linear logit units. The left-hand column locates the participant’s ability along the variable. Each “#” indicates 4 participants, whereas “.” indicates 1–3 participants. The right-hand column locates the items’ difficulty measures along the variable. From the bottom, measures indicate “less satisfaction” for participants and “lower difficulty to be endorsed” for items, whereas “higher satisfaction” and “higher difficulty to be endorsed” are located at the top. The mean difficulty of items in the test is set at 0 logits (and indicated with M). Hence, a candidate with mean ability is indicated by M′. The two arrows represent the threshold boundaries for item difficulty.

## 4. Discussion

This study aimed to assess the psychometric properties of the Arabic version of the PROMIS GLS SF5a in general adult populations in Saudi Arabia. The findings showed that the scale exhibited a unidimensional structure with no item dependence; moreover, with the exception of one item, all items demonstrated a favourable fit to the Rasch model. Consequently, the item separation reliability and internal consistency were excellent, and the person separation reliability was deemed good. Internal consistency was rated as excellent. 

However, the rating scales did not meet the criteria for category functioning because of item disorder. In the Rasch analysis, ‘disordered thresholds’ refer to a situation where the response categories (or thresholds) for an item in a measurement instrument do not function as expected [23]. These thresholds represent the points on the measurement scale where respondents change from one response category to the next, indicating increasing levels of the measured trait or ability. When thresholds are disordered, the responses do not follow the expected pattern of increasing difficulty or severity. For instance, respondents may not consistently choose higher response categories (indicating higher levels of trait or ability) as the item becomes more difficult. Instead, they might occasionally choose a lower response category despite having a higher level of the measured trait or vice versa. This can lead to a lack of discrimination between respondents at different ability levels, thereby reducing the reliability and validity of the measurement instrument [23].

The observed disorder in the rating scale categories can be attributed to a combination of factors. One contributing factor may be the presence of too many response options, specifically seven categories. When participants are presented with such a wide range of choices, it can lead to confusion and difficulty in accurately selecting the most appropriate response, resulting in item disordering [28]. Furthermore, the inclusion of response option 4, *neither agree nor disagree*, within the seven-category scale may be indicative of a fundamental problem. This response option suggests that the scale with seven categories might not be the most suitable choice for accurately capturing respondents’ perceptions or attitudes. The presence of this midpoint option implies that respondents may often find themselves in a state of ambivalence or uncertainty, which can hinder the precision of the data collected [28]. Therefore, it is crucial to reconsider the quantity of response categories in the rating scale. A more suitable approach might be to simplify the scale to a fewer number of categories, which can enhance respondents’ ease of use and improve the overall quality of the data collected. 

The unidimensionalityof the GLS SF5a was confirmed via principal component analysis (PCA), which revealed the absence of a second dimension. In addition, the analysis affirmed the local independence of the items. Furthermore, the reliability indices indicated robust performance, with a high person separation index (2.45), substantial person reliability (0.86) and Cronbach’s α coefficient of 0.90. Notably, our results (Cα = 0.90) closely aligned with those reported by Vaughan et al. [11], consistent with the reliability of the GLS SF5a English version (Cα = 0.91), underscoring the scale’s enhanced precision. In the context of fit statistics analysis, it is noteworthy that item PA046, ‘If I could live my life over, I would change almost nothing’ slightly underfit the scale. This observation signifies a heightened variability in responses for this particular item. The misfitting values were primarily associated with four healthy participants—despite a substantial agreement about all the other items— who expressed disagreement about their overall satisfaction with how they live their lives. 

The item difficulty revealed that the questionnaire’s targeting was acceptable, with a person’s mean ability of 0.29 logits. Item PA046 “If I could live my life over, I would change almost nothing” was the hardest to endorse, indicating less agreement “satisfaction” with the item and lower scores, while item PA047 “I am satisfied with my life” was the easiest to endorse, indicating a high level of agreement or “satisfaction” with this item, consequently yielding higher scores. It is important to note that direct comparisons with previous studies are not feasible, as no prior research has assessed item difficulty within the context of the GLS SF5. 

The Saudi general population had a mean *t*-score of 48.52 ± 10.53, which is comparable to the mean *t*-score of the US GP (50 ± 10), suggesting a similar level of satisfaction between the two populations [22]. Care should be taken when interpreting the results and generalising them to different contexts, as several limitations were observed in the current study. Although data were collected from multiple centres, they were all in the same city; therefore, to improve generalisation, data from multiple cities is recommended. Furthermore, the sex distribution among participants was considered a limitation of this study, as the percentage of female participants was higher than that of male participants (Table 1). It is imperative to acknowledge the disparities observed in the psychological domain, and these findings should be duly recognised as notable limitations. Future studies should evaluate different forms of responses to enhance the options’ interpretability and the scale’s feasibility. Moreover, we recommend applying the Arabic version of the PROMIS GLS SF5a to specific disease populations to allow for better and more specific comparisons with previous studies.

## 5. Conclusions

The psychometric properties of the Arabic version of the PROMIS GLS SF5a scale are promising for measuring general life satisfaction among Saudi Arabia’s general and clinical-based populations. Its application holds promise for efficiently providing high-quality care to patients and for guiding practitioners in tailoring appropriate care plans. Future research should assess its responsiveness, interpretability and feasibility while refining the measures to address the limitations encountered in this study. 

## Figures and Tables

**Table 1:** The FACITtrans Arabic translation team.

Role	Qualification	Title & Profession
Translation Account Manager FACITtrans	MBA	Director, PROMIS Lead
Translation Project Coordinator FACITtrans	BA	Senior COA Translations Manager—Life Sciences, PROMIS Specialist
Translation Project Manager FACITtrans	BA, Spanish Linguistics	Senior COA Translation Project Manager—Life Sciences
Forward 1	BA, Languages and Translation, Simultaneous Interpretation (English < >Arabic)	Senior Translator, Copywriter & Proofreader Professional and Translator Interpreter
Forward 2	MA, Linguistics	Professional Translator and Interpreter
Reconciler/Proofreader	Ph.D., Linguistics	Professional Linguist and Translator
Back Translator	MA, Diplomacy BA, Medical Technology	Professional Translator 16 years full immersion in Arabic public school system and 3.5 years of undergraduate education (nursing) at King Abdul Aziz University, Jeddah Saudi Arabia
Reviewer 1	Ph.D., Linguistics	Linguist and researcher Pragmatics, sociolinguistics, discourse analysis, ideology, identity, and translation studies
Reviewer 2	Ph.D., Linguistics	Professional Linguist and Translator
Reviewer 3, Language Coordinator, Proofreader 1	DDS MA, Biblical Studies	Professional Translator and Interpreter Close to 30 years’ experience specializing in medical, legal and religious translation

**Table 2:** Characteristics of the sample (*n* = 657).

Variables	Total (*n* = 657)
Age in years (Mean ± SD)	28.44 ± 11.36
BMI (Mean ± SD)	24.75 ± 5.76
**Sex *n* (%)**	
Female	534 (81.28%)
Male	123 (18.72%)
**Educational level** ***n* (%)**	
Elementary	6 (0.91%)
Middle School	3 (0.46%)
High School	90 (13.70%)
Diploma	12 (1.83%)
Bachelors	485 (73.82%)
Graduate studies	61 (9.28%)
**Employment status** ***n* (%)**	
Student	287 (43.68%)
Governmental	196 (29.83%)
Private sector	21 (3.20%)
Military	3 (0.46%)
Retired	12 (1.83%)
Unemployed	138 (21%)
**Marital status *n* (%)**	
Married	176 (26.79%)
Single	462 (70.32%)
Divorced	16 (2.44%)
Widowed	3 (0.46%)
**Presence of chronic condition, *n* (%)**	
Yes	125 (19.03%)
No	532 (80.97%)

**Table 3:** Category functioning for Arabic PROMIS General Life Satisfaction SF5a.

Category Label	Category Measure	Andrich Threshold	Infit MnSq	Outfit MnSq	Observed Count
1. Strongly disagree	−3.34	None	1.46	1.30	332 (10%)
2. Disagree	−1.76	−2.00	0.88	1.03	364 (11%)
3. Slightly disagree	−0.78	−1.17	0.85	0.89	472 (14%)
4. Neither agree nor disagree	−0.10	0.16	0.94	0.90	313 (10%)
5. Slightly agree	0.61	−0.39	0.90	0.86	653 (20%)
6 Agree	1.82	0.93	0.93	0.93	697 (21%)
7. Strongly agree	3.71	2.47	1.08	1.02	454 (14%)

**Table 4:** Item Fit Statistics for PROMIS General Life Satisfaction SF5a.

Item	Measure (SE)	Infit		Outfit	
		MnSq	Zstd	MnSq	Zstd
**If I could live my life over, I would change almost nothing.**	**0.89 (0.04)**	**1.30**	**4.74**	**1.33**	**4.82**
So far, I have gotten the important things I want in life.	0.05 (0.04)	1.03	0.51	1.04	0.65
In most ways, my life is close to perfect.	0.18 (0.04)	0.86	−2.63	0.87	−2.21
My life situation is excellent.	−0.46 (0.04)	0.87	−2.30	0.86	−2.41
My life is just right.	−0.67 (0.04)	0.84	−2.02	0.78	−3.88

Item fit statistics shows the 4 items that fitted the scale with the underfit item measurement in bold.

## Data Availability

The datasets analysed during the current study are available from the corresponding author upon reasonable request.
